# Cannabidiol protects keratinocyte cell membranes following exposure to UVB and hydrogen peroxide

**DOI:** 10.1016/j.redox.2020.101613

**Published:** 2020-06-23

**Authors:** S. Atalay, I. Dobrzyńska, A. Gęgotek, E. Skrzydlewska

**Affiliations:** a Department of Analytical Chemistry, Medical University of Białystok, Poland; b Institute of Chemistry, University of Białystok, Białystok, Poland

**Keywords:** Keratinocyte cell membrane, Cannabidiol, UVB, Hydrogen peroxide, Oxidative stress, Antioxidative defense

## Abstract

Keratinocytes, the major cell type of the epidermis, are particularly sensitive to environmental factors including exposure to sunlight and chemical agents. Since oxidative stress may arise as a result of these factors, compounds are actively sought that can act as protective agents. Recently, cannabidiol (CBD), a phytocannabinoid found in *Cannabis Sativa* L., has gained increased interest due to its anti-inflammatory and antioxidant properties, and absence of psychoactive effects. This prompted us to analyze the protective effects of CBD on keratinocytes exposed to UVB irradiation and hydrogen peroxide. Here we show, using liquid chromatography mass spectrometry, that CBD was able to penetrate keratinocytes, and accumulated within the cellular membrane. CBD reduced redox balance shift, towards oxidative stress, caused by exposure UVB/hydrogen peroxide, estimated by superoxide anion radical generation and total antioxidant status and consequently lipid peroxidation level. CBD was found to protect keratinocytes by preventing changes in the composition of the cellular membrane, associated with UVB/hydrogen peroxide damages which included reduced polyunsaturated fatty acid levels, increased sialic acid and lipid peroxidation products (malondialdehyde and 8-isoprostanes) levels. This maintains cell membranes integrity and prevents the release of lactate dehydrogenase. In addition, CBD prevented UVB/hydrogen peroxide-induced reduction of keratinocyte size and zeta potential, and also decreased activity of ATP-binding cassette membrane transporters. Together, these findings suggest that CBD could be a potential protective agent for keratinocytes against the harmful effects of irradiation and chemical environmental factors that cause oxidative stress.

## Highlights

• UVB/H_2_O_2_ stimulates keratinocytes membrane penetration by CBD.
• CBD protects cells against UVB/H_2_O_2_ induced redox imbalance.
• CBD maintains membrane integrity by preventing its component modifications.
• CBD decreases activity of ATP-binding cassette membrane transporters.
• CBD could be a potential keratinocytes protector against the harmful factors.

## Graphical abstract

**Figure:**
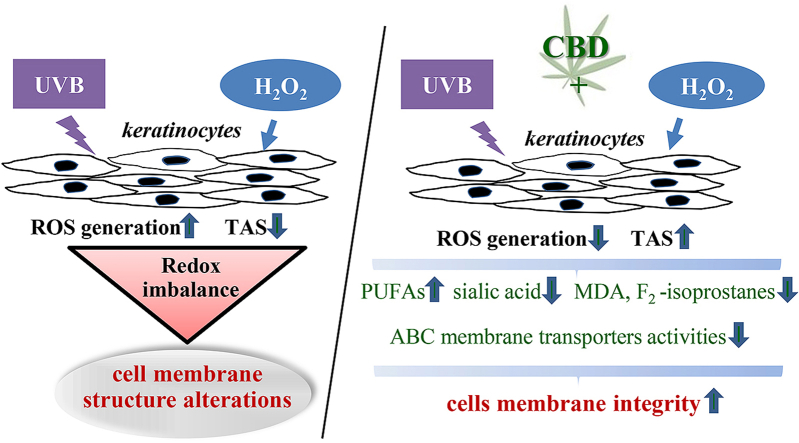

## Abbreviations

4-HNE: 4-hydroxynenenal
8-isoPGF_2a_: 8-isoprostaglandin F_2a_
ABTS: 2,2′-azino-bis-3-ethylbenzthiazoline-6-sulfonic acid
AKT: protein kinase B
BACH1: transcription regulator protein/BRCA1 associated C-terminal helicase
BCRP: breast cancer resistance protein
CBD: cannabidiol
CMH: 1-hydroxy-3-methoxycarbonyl-2,2,5,5-tetramethyl-pyrrolidine
COX2: cyclooxygenase 2
DCBD: decarbonylated cannabidiol
DLS: dynamic light scattering
DMEM: Dulbecco's Modified Eagle's Medium
DMSO: dimethyl sulfoxide
ESI: electrospray ionization source
ESR: electron spin resonance
FABPs: fatty acid-binding proteins
FAMEs: fatty acid methyl esters
FBS: fetal bovine serum
FID: flame ionization detector
GC: gas chromatograph
H_2_O_2_: hydrogen peroxide
HPLC: high-performance liquid chromatography
IFN-β: interferon beta
IFN-γ: interferon gamma
IL-10: interleukin 10
IL-17: interleukin 17
IL-1β: interleukin 1 beta
IL-4: interleukin 4
IL-6: interleukin 6
iNOS: inducible nitric oxide synthase
JNK: Jun N-terminal kinase
LCMS: liquid chromatography–mass spectrometry
LDH: lactate dehydrogenase
LDV: laser doppler velocimetry
LPS: lipopolysaccharide
MDA: malondialdehyde
MDR1: multidrug resistance protein 1
MRM: multiple-reaction monitoring
MRP: multidrug resistance proteins
NADPH: nicotinamide adenine dinucleotide phosphate
NF-κB: nuclear factor-κB
NOX: NADPH oxidase
Nrf2: nuclear factor erythroid 2-related factor 2
OATP: organic-anion-transporting polypeptide
p38: p38 mitogen-activated protein kinase
p53: cellular tumor antigen/tumor-suppressor protein
PUFAs: polyunsaturated fatty acids
ROS: reactive oxygen species
SPE: solid phase extraction
TAS: total antioxidant status
TLC: thin-layer chromatography
TNF-α: tumor necrosis factor alpha
VDACs: voltage-dependent anion channels
XO: xanthine oxidase

## 1 Introduction

The skin is the primary organ protecting the human body against the damaging effects of exogenous factors, and also participates in maintaining internal homeostasis [1]. As a result, skin cells, especially epidermal cells, are constantly exposed to both irradiation (including daily ultraviolet, UV) and chemical agents that contribute to increased production of reactive oxygen species (ROS) and impaired endogenous antioxidant efficacy [2,3]. This leads to redox imbalance and the formation of oxidative stress [4]. The skin cells most sensitive to environmental factors are keratinocytes, which are the basic cells that build the epidermal layer [5].

The cell membranes of keratinocytes, the basic components of which are phospholipids, are the cellular part most exposed to environmental factors. In the event of disruption of redox homeostasis in cells, ROS act as transient signaling molecules [6] and stimulate the production of further lipid mediators, such as lipid peroxidation products [7]. Lipid peroxidation occurs as the result of nonenzymatic and enzymatic processes, and the most sensitive peroxidation molecules are free or phospholipid polyunsaturated fatty acids (PUFAs), including arachidonic, linoleic, linolenic, eicosapentaenoic and docosahexaenoic acids [8]. ROS may lead to oxidative cyclization of phospholipid PUFAs to form F_2_-isoprostanes or D_2_/E_2_-isoprostanes [9]. Fatty acids released by phospholipases undergo oxidative fragmentation to form α, β-unsaturated aldehydes, including malondialdehyde (MDA), also 4-hydroxynenenal (4-HNE), and other aldehydes [10,11]. The structure and function of biological membranes may be affected by oxidative modifications of membrane proteins [12,13]. Moreover, sialic acid - a component of glycolipids and glycoproteins and a carrier of a negative charge on the surface of the membrane - participates in maintaining the physicochemical properties of cell membranes [[14], [15], [16]].

Modification of the chemical composition of biomembranes, including the creation lipid peroxidation products, disturbs lipid asymmetry. This reduces the hydrophobicity of the interior of the lipid membrane, causing its depolarization. Such changes directly disrupt the proper functioning of the cell membrane, potentially leading to a loss of membrane integrity and an increase in its permeability [17]. Additionally, lipid peroxidation products are chemically reactive molecules due to their chemical structure and consequentially, lipid peroxidation products can easily form adducts with most of the cell's nucleophilic components, including proteins, lipids and DNA, leading to cellular metabolism disorders. These reactions reduce the level of free reactive products of lipid peroxidation, while increasing, for example, the level of adducts of formed aldehydes and proteins (e.g. 4-HNE-protein adducts). Such products promote disorders of cell signaling, and thus stimulate metabolic modifications that lead to cellular dysfunction followed by cell death or tumor transformation [18].

As a result of these processes, research is being conducted to search for compounds (preferably of natural origin) that protect skin cells against the effects of exogenous factors, especially those that disturb the redox balance [19,20]. An example of a recently intensively studied compound is cannabidiol (CBD), one of the major phytocannabinoids found in *Cannabis sativa* L., which has no psychoactive effects [21]. CBD has been shown to be a lipophilic antioxidant with anti-inflammatory properties [22,23]. Observations have shown that treatment of atherosclerotic testicular cells from male Sprague-Dawley rats with CBD causes inhibition of the expression of proxidative and proinflammatory proteins [inducible nitric oxide synthase (iNOS) and cyclooxygenase 2 (COX2)] [24]. CBD treatment also reduced the activity of iNOS activated by lipopolysaccharide (LPS) in mouse macrophages [25]. In addition, CBD has also been found to improve the antioxidant properties of cells by regulating the expression of nuclear factor erythroid 2-related factor 2 (Nrf2) [26,27]. Importantly, CBD possesses neuroprotective capabilities, as an increase in superoxide dismutase mRNA expression was observed in Parkinson's and Huntington's disease models following CBD treatment [23]. This antioxidant/neuroprotective effect has been found to involve the activation of signaling pathways implicated in controlling redox balance, which CBD can restore [28]. The anti-inflammatory effects of CBD have been highlighted in the mouse model of multiple sclerosis, where CBD therapy lowered the level of proinflammatory cytokines such as interleukin 1 beta (IL-1β), tumour necrosis factor alpha (TNF-α), interferon beta (IFN-β), interferon gamma (IFN-γ), interleukin 17 (IL-17) and interleukin 6 (IL-6), and increased the levels of anti-inflammatory cytokines [interleukin 4 (IL-4) and interleukin 10 (IL-10)] [23]. Additionally, in a mouse model of diabetic cardiomyopathy, CBD relieved oxidative/nitrosative stress and inflammation, and protected related cell signaling pathways [29].

Given the currently available data, the purpose of this work was to assess the impact of CBD on the structure and function of the cell membranes of keratinocytes exposed to oxidative stress. UVB radiation and hydrogen peroxide were used as physical and chemical agents, respectively.

## 2 Material and methods

### 2.1 Cell culture and treatment

Human keratinocytes (CDD 1102 KERTr) obtained from American Type Culture Collection were cultured in Dulbecco's Modified Eagle's Medium (DMEM) with 10% fetal bovine serum (FBS) supplemented with 50 U/mL penicillin and 50 μg/mL streptomycin. The cells were cultured in a humidified atmosphere of 5% CO_2_ at 37 °C up to 70% confluence. After reaching the required confluency, keratinocytes were divided into 3 main groups:

#### 2.1.1 Control groups

I. Control cells cultured in standard medium;
II. Cells cultured in medium containing 4 μM CBD (Sigma-Aldrich, MO, USA) in 0.2% ethanol for 24 h;
III. Cells cultured in medium containing 4 μM CBD for 48 h.

#### 2.1.2 UVB-irradiated groups

IV. Cells exposed to UVB irradiation (312 nm) at 60 mJ/cm^2^ (Bio-Link Crossliner BLX 312; Vilber Lourmat, Germany; 6 lamps at a distance of 15 cm);
V. Cells cultured for 24 h after UVB irradiation in medium containing 4 μM CBD;
VI. Cells cultured for 24 h before and 24 h after UVB irradiation in medium containing 4 μM CBD.

#### 2.1.3 Hydrogen peroxide-exposed groups

VII. Cells cultured for 24 h in medium containing 200 μM hydrogen peroxide (this concentration corresponded to 70% cell viability);
VIII. Cells cultured for 24 h after hydrogen peroxide exposure in medium containing 4 μM CBD.
IX. Cells cultured for 24 h before and for 24 h after hydrogen peroxide exposure in medium containing 4 μM CBD.

All results were normalized to the total level of protein measured using Bradford reagent [30].

### 2.2 The effect of CBD on redox state in keratinocytes

#### 2.2.1 Oxidative state

NADPH oxidase [NOX - EC 1.6.3.1] activity was analyzed with the method used by Griendling [31]. Lucigenin was used as a luminophore for the luminescence measurement to determine enzyme-specific activity in relative luminescence units (RLU) per milligram of protein. Xanthine oxidase activity [XO - EC1.17.3.2] was determined using the method of Lin [32], through assessing uric acid formation from xanthine. Specific enzyme activity is given in microunits per milligram of protein.

Superoxide anion generation was analyzed using electron spin resonance (ESR) spectrometer e-scan (Noxygen GmbH/Bruker Biospin GmbH, Germany). This method evaluated the selective interaction of the spin probe 1-hydroxy-3-methoxycarbonyl-2,2,5,5-tetramethyl-pyrrolidine (CMH) (200 μM) with ROS, forming a stable CM nitroxide radical with half-life 4 h. Superoxide anions were measured based on the kinetics of nitroxide accumulation in accordance with the ESR amplitude of the low ESR spectral component, as in a study from Kuzkaya [33]. Superoxide anion generation is expressed in nanomoles per minute per milligram of protein.

#### 2.2.2 Antioxidative state

Total antioxidant status (TAS) was determined using 2,2′-azino-bis-3-ethylbenzthiazoline-6-sulfonic acid (ABTS) [34]. A solution of cationic ABTS radicals was prepared in phosphate buffer (PBS, pH 7.4). 3-fold diluted samples (5 μl) were mixed with ABTS radical working solution (245 μl) in a 96 well plate at 37 °C for 10 min. Absorbance at 734 nm was then measured using an Infinite 200 plate reader (TEKAN, Switzerland). Ultimately, TAS is expressed as mg vitamin C/mg protein.

### 2.3 Characteristics of keratinocyte membrane

#### 2.3.1 Phospholipid fatty acid composition

Phospholipid fatty acids profiles of keratinocyte membranes were determined using gas chromatography (GC) according to the method of Christie [35]. Lipid components were isolated using chloroform/methanol mixture (2:1, v/v) (Folch extraction) in the presence of 0.01% butylated hydroxytoluene–BHT. Using thin-layer chromatography (TLC), phospholipid fatty acids were separated using as mobile phase mixture of heptane-diisoprophyl ether-acetic acid (60:40:3,v/v/v). Next, fatty acids were transmethylated to fatty acid methyl esters (FAMEs) with boron trifluoride in methanol under nitrogen atmosphere at 100 °C for 30 min. FAMEs were analyzed by GC/flame ionization detector (FID) on Clarus 500 Gas Chromatograph (PerkinElmer, MA, USA). The separation of FAMEs was performed on a capillary column coated with Varian CP-Sil88 stationary phase (50 m × 0.25 mm, ID 0.2 μm, Varian). Operating conditions were as follows: the split-splitless injector was used in split mode (split ratio of 1:20); the injection volume of the sample was 2 μL; the temperature of the injector and detector was 260 °C while column temperature was programmed from 150 °C (2 min) to 230 °C (10 min) at 4 °C/min; finally, the carrier gas used was helium (flow rate of 1 ml/min). Identification of FAMEs was performed by comparison retention time using an internal standard (1,2-dinonadecanoyl-sn-glycero-3-phosphocholine (19:0 PC) for phospholipid fatty acids). The phospholipid fatty acid concentration was expressed as a micrograms per milligram of protein.

#### 2.3.2 Sialic acid content

Total sialic acid content was determined by modified Jourdian's resorcinol method [36]. Color intensity was measured at 630 nm using a diode array spectrophotometer (Hewlett Packard). The sialic acid level was read from the standard curve for the N-acetylneuraminic acid solution (31.25–1000 nmol/ml) and normalized for milligrams of protein. Results are expressed as a percentage of sialic acid level in control cells.

#### 2.3.3 Lipid peroxidation products

Malondialdehyde (MDA) was measured as malondialdehyde-thiobarbituric acid adducts by high-performance liquid chromatography (HPLC) with spectrofluorometric detection (*λ*_ex_ = 532 nm/*λ*_em_ = 553 nm) [37]. After protein precipitation and removing MDA adducts (centrifugation at 10.000×*g* for 15 min at 4 ^°^C) with thiobarbituric acid (42 mM) were analyzed. After 60 min incubation at 100 °C, samples were mixed with MetOH-NaOH (1:1) in vials. HPLC separation of MDA-TBA adducts was followed with the mobile phase consisting of 40:60 (v/v) methanol: potassium phosphate buffer (0.05 M, pH 6.8) on RP C18 column. The MDA concentration was determined using a calibration curve range: 1–120 nmol/L (r^2^- 0.9995) and expressed as micromoles per milligram of protein.

F_2_-isoprostanes (8-isoPGF_2a_) were measured by liquid chromatography-mass spectrometry (LCMS) (Nexera X2 ultra high performance liquid chromatograph interfaced with LCMS-8060, Shimadzu, Kyoto, Japan) with electrospray ionization source (ESI) operating negative ion mode with multiple-reaction monitoring (MRM) using the method reported by Coolen [38]. In LC-MS analysis with negative-ion mode, 8-isoPGF_2a-d4_ was used as an internal standard and mass transition was analyzed as 353.2 → 193.1 for 8-isoPGF_2a_, 357.2 → 197.1 for 8-isoPGF_2a-d4_. The limit of detection was 1 pg/ml. Results were normalized for milligrams of protein and expressed as pg/mg protein.

#### 2.3.4 Cell size and zeta potential

Changes in phospholipid composition of the cell membrane affect physicochemical properties of cells such as cells size and membrane zeta potential. To determine these parameters, keratinocytes were suspended in PBS and placed in a measuring vessel to determine cell size and zeta potential of the cell membrane. These parameters were measured using a Zetasizer Nano ZS apparatus (Malvern Instruments, UK). This apparatus uses a process called Dynamic Light Scattering (DLS) and Laser Doppler Velocimetry (LDV).

#### 2.3.5 Membrane permeability

Lactate dehydrogenase (LDH) leakage into medium was used as a measure of plasma membrane permeabilization [39]. The activity of LDH in the medium or cell lysates was estimated by the NADH level decrease in the presence of pyruvate measured spectrophotometrically at 340 nm (Multiskan GO Microplate Spectrophotometer Thermo Scientific, USA). The percentage LDH release from cells was calculated by comparing activity in the medium to the cell lysate.

#### 2.3.6 CBD cytosolic and membrane concentrations

Cytosol and membrane CBD level in keratinocytes was determined using ultra-performing liquid chromatography tandem mass spectrometry (LCMS 8060, Shimadzu, Kioto, Japan) using a recently published method [40]. CBD was extracted using solid phase extraction (SPE) and analyzed in positive-ion mode. CBD-d_9_ was used as an internal standard for quantification. The precursor to the product ion transition was 315.1 → 193.00 for CBD. The results were normalized for milligrams of protein and are expressed as μg/mg protein.

#### 2.3.7 Transmembrane OATP transporter activity

The activity of OATP transporters were determined using a Multidrug-Resistance assay according to the manufacturer's protocols (eFluxx-ID Multidrug resistance assay kits, Enzo LifeSciences, UK). Keratinocytes with inhibitors of OATP transporters [multidrug resistance protein 1 (MDR1), multidrug resistance-associated proteins (MRP), breast cancer resistance protein (BCRP)] in DMSO, and keratinocytes without inhibitors (containing PBS and DMSO) were incubated in dark 96 well plates for 5 min (37 °C). Next, EFLUXX-ID® green detection reagent was added, and samples were incubated for 30 min (37 °C). Luminescence was measured [λ_ex_485 nm/λ_em_535 nm] using EnSpire 2300 Multilable Reader (PerkinElmer, MA, USA). Activities of MDR1, MRP, BCRP were normalized to the total level of protein, and the final results were expressed as a percentage of the activity of transporters compared to control cells.

### 2.4 Statistics

Data were analyzed using standard statistical analyses, including one-way ANOVA for multivariate analyses. Results are expressed as mean ± standard deviation (SD) for n = 5. P values less than 0.05 were considered significant.

## 3 Results

### 3.1 Effect of CBD on keratinocytes redox status

**Fig. 1:**
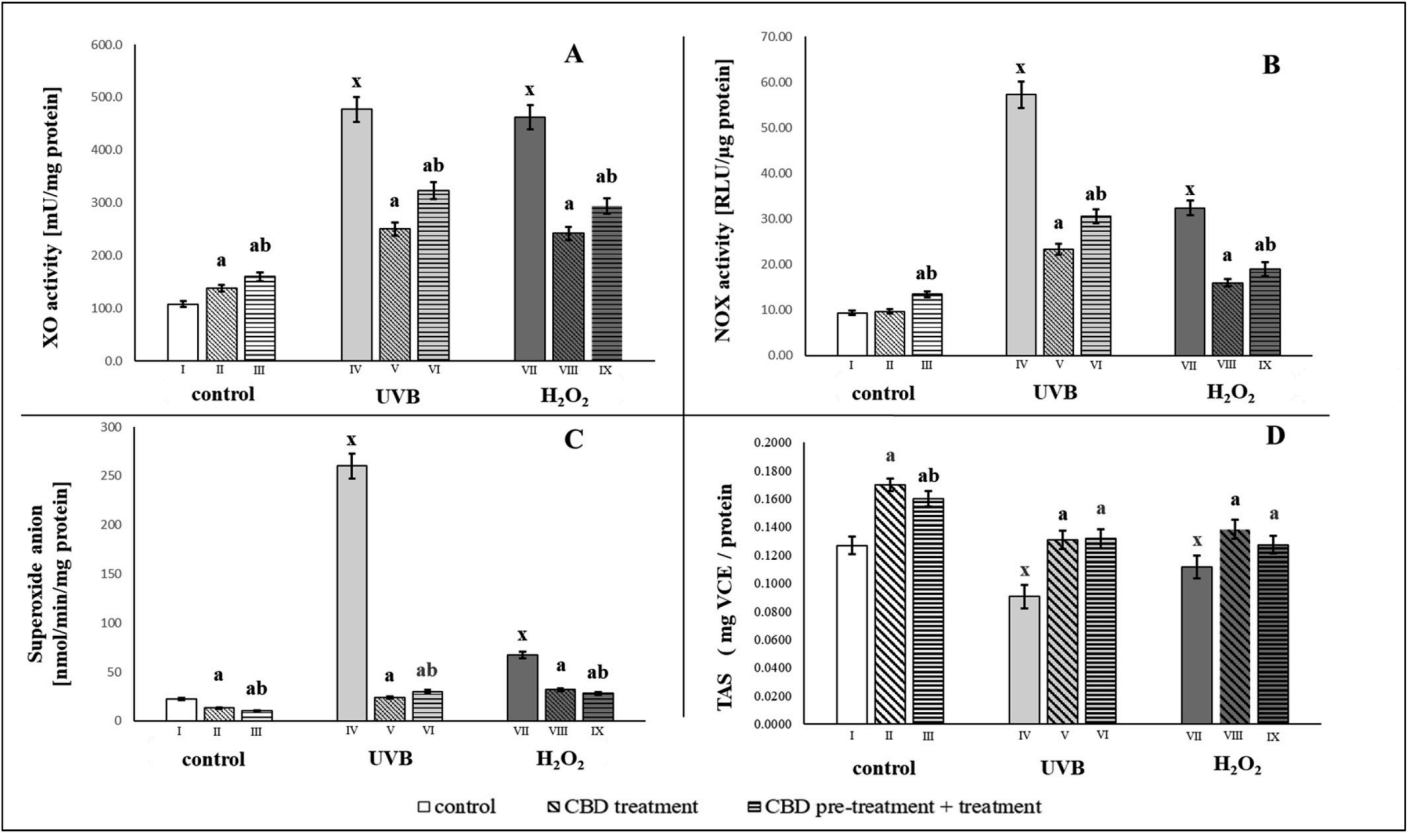
The redox status estimated through pro-oxidant enzymes activity (XO - A; NOX - B), superoxide anion generation (C) and Total Antioxidant Status (D) in groups of keratinocytes: **I.** control; **II.** cultured with CBD (4uM) for 24h; **III**. cultured with CBD (4uM) for 48h; **VI.** irradiated with UVB [60 mJ/cm^2^]; **V.** irradiated with UVB [60 mJ/cm^2^] and cultured with CBD (4uM) for 24h after irradiation; **VI.** irradiated with UVB [60 mJ/cm^2^] and cultured with CBD for 24h befor irradiation and 24h after irradiation; **VII.** exposed to H_2_O_2_ [200μM]; **VIII.** exposed to H_2_O_2_ [200μM] and cultured with CBD (4uM) for 24h after exposer to H_2_O_2_; **IX.** exposed to H_2_O_2_ [200μM] and cultured with CBD (4uM) for 24h before and 24h after exposer to H_2_O_2_. Mean values ± SD of five independent experiments and statistically significant differences for p < 0.05 are presented: a - differences vs. group I/group IV/group VII; b - differences vs. group II/group V/group VIII; x - differences between group IV/group VII and group I.

To assess the effects of CBD on redox conditions in keratinocytes, the cells were exposed to UVB irradiation or hydrogen peroxide before measuring XO and NOX activity, superoxide anion generation and total antioxidant status (TAS). After exposure of the cells to UVB irradiation and hydrogen peroxide, keratinocytes showed a 4-fold increase in xanthine oxidase activity, while CBD treatment was followed by a 2-fold decrease in the activity versus mentioned increased activity (Fig. 1). NADPH oxidase activity after UVB irradiation increased approximately 6-fold, and after the use of hydrogen peroxide increased 3-fold. Exposure of the keratinocytes to CBD reduced the NADPH oxidase activity by approximately 50%. As a result, the level of superoxide anions production also decreased after CBD treatment. In cells pretreated and/or treated with CBD, production of UVB-induced superoxide anions was reduced by approximately 90%, while in hydrogen peroxide-treated cells, pretreatment and/or treatment of keratinocytes with CBD led to a reduction in the level of superoxide anion production by almost 50% (Fig. 1).

CBD antioxidant activity was also revealed by its effect on the total status of keratinocyte antioxidants (TAS), which increased significantly after CBD treatment in all treated groups (Fig. 1D).

#### 3.1.1 Effect of CBD on keratinocyte membrane composition

The effect of CBD on the levels of phospholipid PUFAs, sialic acid and lipid peroxidation products was measured after keratinocytes exposure to UVB irradiation and hydrogen peroxide. The results indicated that the level of phospholipid polyunsaturated fatty acids (PUFAs), especially arachidonic and docosahexaenoic acid, dropped dramatically (by about 65%) in UVB irradiated cells (Table 1). The exposure of keratinocytes to hydrogen peroxide led to an increase in all phospholipid fatty acids. Treatment of keratinocytes with CBD before and after UVB irradiation increased the level of phospholipid PUFAs by 3-fold. The level of docosahexaenoic acid was much higher in cells treated with CBD before and after UVB irradiation compared to cells treated only after UVB irradiation. The use of CBD after hydrogen peroxide exposure resulted in a reduction of approximately 20% in the level of linoleic, γ-linolenic, arachidonic and eicosapentaenoic acid. On the other hand, keratinocytes treated with CBD before and after hydrogen peroxide had a further reduced level of these fatty acids (linoleic acid by about 30%; arachidonic and docosahexaenoic acid by 17%; γ-linolenic, oleic, linolenic and eicosapentaenoic acid by approximately 25%). Consequently, cells treated with CBD after hydrogen peroxide generally had higher levels of fatty acids (linolenic, linoleic, oleic, γ-linolenic and arachidonic) than cells treated with CBD before and after exposure to hydrogen peroxide. The results indicate that CBD did indeed partially prevent redox imbalance and its consequences in keratinocyte membranes despite exposure to exogenous factors.

**Table 1:** Unsaturated phospholipid fatty acids profiles in groups of keratinocytes: **I.** control; **II.** cultured with CBD (4uM) for 24h; **III**. cultured with CBD (4uM) for 48h; **VI.** irradiated with UVB [60 mJ/cm^2^]; **V.** irradiated with UVB [60 mJ/cm^2^] and cultured with CBD (4uM) for 24h after irradiation; **VI.** irradiated with UVB [60 mJ/cm^2^] and cultured with CBD for 24h befor irradiation and 24h after irradiation; **VII.** exposed to H_2_O_2_ [200μM]; **VIII.** exposed to H_2_O_2_ [200μM] and cultured with CBD (4uM) for 24h after exposer to H_2_O_2_; **IX.** exposed to H_2_O_2_ [200μM] and cultured with CBD (4uM) for 24h before and 24h after exposer to H_2_O_2_. Mean values ± SD of five independent experiments and statistically significant differences for p < 0.05 are presented:a - differences vs. group I/group IV/group VII; b - differences vs. group II/group V/group VIII; x - differences between group IV/group VII and group I.

	Control groups			Keratinocytes treated with UVB			Keratinocytes treated with H_2_O_2_		
Fatty acids μg/mg of protein	Control (I)	CBD treatment (II)	CBD pretreatment + treatment (III)	UVB (IV)	CBD treatment (V)	CBD pretreatment + treatment (VI)	H_2_O_2__(VII)_	CBD treatment (VIII)	CBD pretreatment + treatment (IX)
Oleic (C18:1n9c)	69.2 ± 3.4	80.6 ± 4.0^**a**^	77.7 ± 3.9^**a**^	23.4 ± 1.2^**x**^	67.7 ± 3.4^**ab**^	77.3 ± 3.9^**ab**^	82.6 ± 4.1^**x**^	77.1 ± 3.8	70.7 ± 3.5^**a**^
Linoleic (C18:2n6c)	21.6 ± 1.1	25.3 ± 1.3^**a**^	22.5 ± 1.1^**b**^	7.9 ± 0.4^**x**^	22.4 ± 1.1^**a**^	18.4 ± 0.9^**ab**^	28.2 ± 1.1^**x**^	22.1 ± 1.1^**a**^	18.9 ± 0.9^**ab**^
γ-Linolenic (C18:3n6)	7.44 ± 0.37	8.86 ± 0.44^**a**^	7.16 ± 0.36^**b**^	2.70 ± 0.13^**x**^	7.57 ± 0.38^**a**^	6.89 ± 0.34^**a**^	10.13 ± 0.51^**x**^	8.40 ± 0.42^**a**^	7.23 ± 0.36^**ab**^
Linolenic (C18:3n3)	0.10 ± 0.01	0.12 ± 0.01	0.11 ± 0.01	0.03 ± 0.01^**x**^	0.09 ± 0.01^**a**^	0.11 ± 0.01^**ab**^	0.16 ± 0.01^**x**^	0.13 ± 0.01^**a**^	0.11 ± 0.01^**a**^
Arachidonic (C20:4n6)	8.48 ± 0.42	9.98 ± 0.50^a^	9.15 ± 0.46	2.97 ± 0.15^x^	8.61 ± 0.43^a^	8.72 ± 0.44^a^	10.22 ± 0.51^x^	9.11 ± 0.46^a^	8.10 ± 0.40^ab^
*cis*-5,8,11,14,17-Eicosapentaenoic (C20:5n3)	4.40 ± 0.22	5.16 ± 0.26^a^	4.44 ± 0.22^b^	1.51 ± 0.08^x^	4.30 ± 0.22^a^	4.50 ± 0.22^a^	5.33 ± 0.27^x^	4.64 ± 0.23^a^	4.23 ± 0.21^a^
*cis*-4,7,10,13,16,19-Docosahexaenoic (C22:6n3)	2.60 ± 0.13	3.23 ± 0.16^a^	3.05 ± 0.15^a^	0.91 ± 0.05^x^	2.67 ± 0.13^a^	3.11 ± 0.16^ab^	3.20 ± 0.16^x^	3.00 ± 0.15	2.84 ± 0.14^a^

**Fig. 2:**
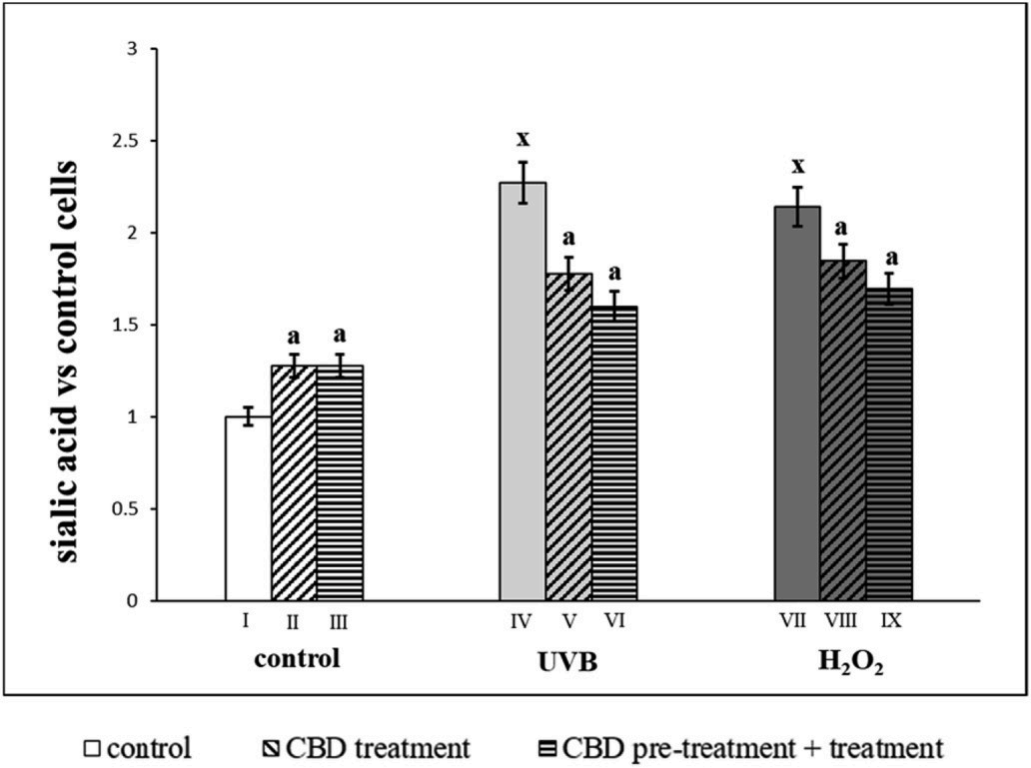
Sialic acid level in groups of keratinocytes: **I.** control; **II.** cultured with CBD (4uM) for 24h; **III**. cultured with CBD (4uM) for 48h; **VI.** irradiated with UVB [60 mJ/cm^2^]; **V.** irradiated with UVB [60 mJ/cm^2^] and cultured with CBD (4uM) for 24h after irradiation; **VI.** irradiated with UVB [60 mJ/cm^2^] and cultured with CBD for 24h befor irradiation and 24h after irradiation; **VII.** exposed to H_2_O_2_ [200μM]; **VIII.** exposed to H_2_O_2_ [200μM] and cultured with CBD (4uM) for 24h after exposer to H_2_O_2_; **IX.** exposed to H_2_O_2_ [200μM] and cultured with CBD (4uM) for 24h before and 24h after exposer to H_2_O_2_. Mean values ± SD of five independent experiments and statistically significant differences for p < 0.05 are presented:a - differences vs. group I/group IV/group VII; b - differences vs. group II/group V/group VIII; x - differences between group IV/group VII and group I.

As well as membrane phospholipids, chemical exposure and irradiation cause changes in the level of sialic acid (Fig. 2), which is one of the basic components of cell membrane glycoproteins and glycolipids. Keratinocytes irradiated by UVB or exposed to hydrogen peroxide, had approximately 2 times higher sialic acid levels than control cells. However, CBD treatment enhanced sialic acid levels in control cells, and decreased the levels to a similar degree in keratinocytes irradiated with UVB or exposure to hydrogen peroxide. Changes in sialic acid levels did not depend on whether CBD was used only after or before and after irradiation/exposure.

**Fig. 3:**
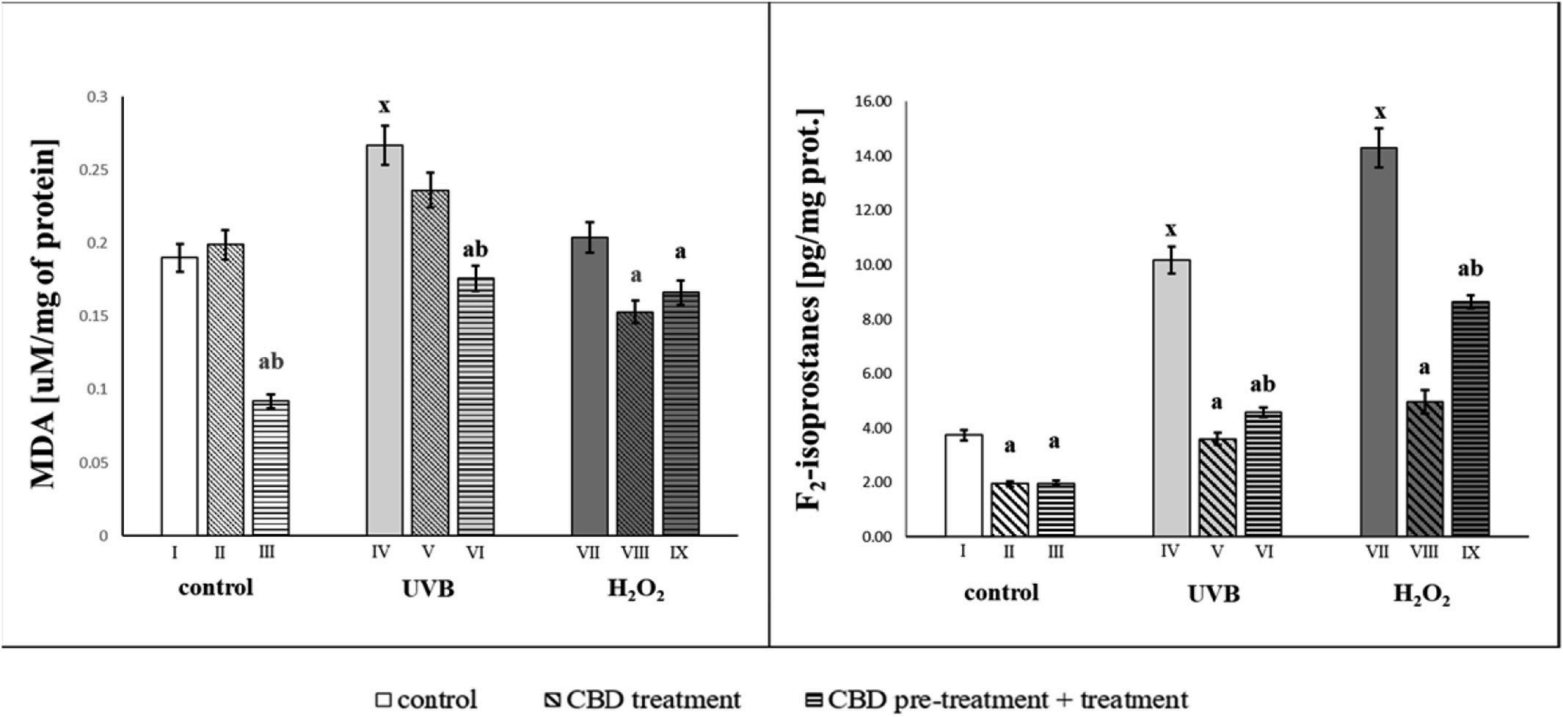
Malondialdehyde (MDA) and F_2_-isoprostanes level in groups of keratinocytes: **I.** control; **II.** cultured with CBD (4uM) for 24h; **III**. cultured with CBD (4uM) for 48h; **VI.** irradiated with UVB [60 mJ/cm^2^]; **V.** irradiated with UVB [60 mJ/cm^2^] and cultured with CBD (4uM) for 24h after irradiation; **VI.** irradiated with UVB [60 mJ/cm^2^] and cultured with CBD for 24h befor irradiation and 24h after irradiation; **VII.** exposed to H_2_O_2_ [200μM]; **VIII.** exposed to H_2_O_2_ [200μM] and cultured with CBD (4uM) for 24h after exposer to H_2_O_2_; **IX.** exposed to H_2_O_2_ [200μM] and cultured with CBD (4uM) for 24h before and 24h after exposer to H_2_O_2_. Mean values ± SD of five independent experiments and statistically significant differences for p < 0.05 are presented:a - differences vs. group I/group IV/group VII; b - differences vs. group II/group V/group VIII; x - differences between group IV/group VII and group I.

The shift in redox balance towards the oxidant reaction under the influence of UVB and hydrogen peroxide is accompanied by intensified ROS-dependent metabolism of PUFAs. This confirms the increase in the level of lipid peroxidation products such as MDA, formed during oxidative fragmentation, as well as F_2_-isoprostanes being the product of oxidative cyclization. The strongest increase in MDA level (1.4 times) was observed after UVB irradiation, while hydrogen peroxide exposure led to an increase in MDA level, but not to a statistically significant degree. CBD prevented UVB- and hydrogen peroxide-induced increases in lipid peroxidation products, including MDA and F_2_-isoprostanes (Fig. 3). CBD treated cells both, before as well as before and after UVB irradiation led to a significant reduction in MDA level, but the treatment of CBD before and after UVB irradiation was more effective. However in CBD-treated keratinocytes both before as well as before and after hydrogen peroxide exposure MDA levels were decreased. Additionally, the level of F_2_-isoprostanes in UVB-irradiated or hydrogen peroxide-exposed keratinocytes was significantly increased, whereas CBD treatment led to a significant decrease in the level of F_2_-isoprostanes. Protective use of CBD in both cases promoted the prevention of oxidative stress, with a significant reduction (50%) of F_2_-isoprostanes level in cells treated with CBD (Fig. 3).

**Fig. 4:**
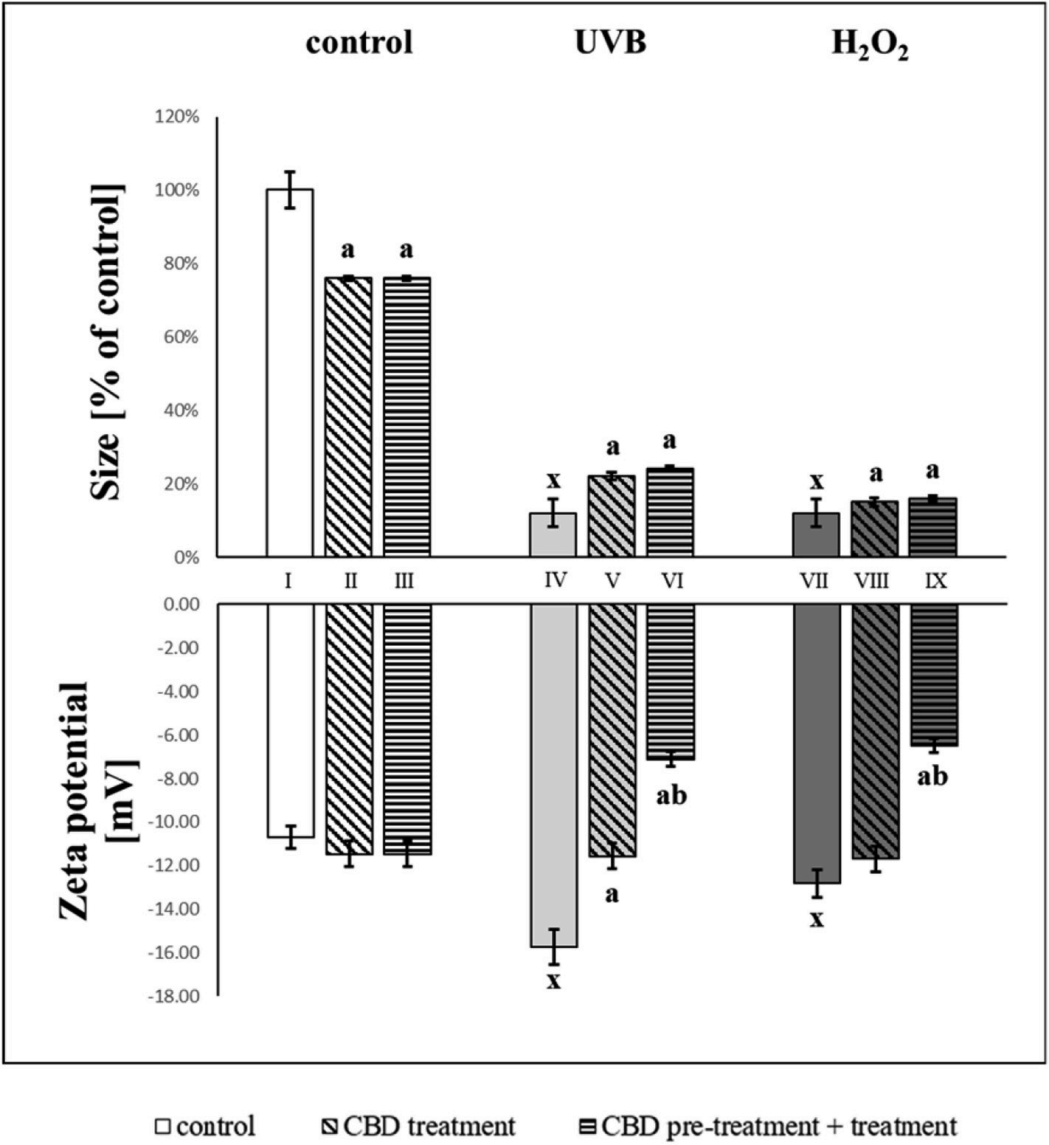
Size [% of control cells] and zeta potential [mV] in groups of keratinocytes: **I.** control; **II.** cultured with CBD (4uM) for 24h; **III**. cultured with CBD (4uM) for 48h; **VI.** irradiated with UVB [60 mJ/cm^2^]; **V.** irradiated with UVB [60 mJ/cm^2^] and cultured with CBD (4uM) for 24h after irradiation; **VI.** irradiated with UVB [60 mJ/cm^2^] and cultured with CBD for 24h befor irradiation and 24h after irradiation; **VII.** exposed to H_2_O_2_ [200μM]; **VIII.** exposed to H_2_O_2_ [200μM] and cultured with CBD (4uM) for 24h after exposer to H_2_O_2_; **IX.** exposed to H_2_O_2_ [200μM] and cultured with CBD (4uM) for 24h before and 24h after exposer to H_2_O_2_. Mean values ± SD of five independent experiments and statistically significant differences for p < 0.05 are presented: a - differences vs. group I/group IV/group VII; b - differences vs. group II/group V/group VIII; x - differences between group IV/group VII and group I.

Changes in the composition of membrane phospholipids are accompanied by modifications in the size of keratinocytes. Both UVB and hydrogen peroxide caused up to an 8-fold reduction in cell size (Fig. 4). However, exposure of control keratinocytes to CBD also resulted in a decrease in cell size, of approximately 24%. CBD was partially protective against damage caused by UVB irradiation and hydrogen peroxide exposure, which was observed as an increase in the volume of cells relative to untreated cells exposed to UVB irradiation.

Due to alterations in membrane phospholipid composition and cell size, changes in zeta potential were also observed (Fig. 4). Negative zeta potential, following UVB or hydrogen peroxide, increased remarkably compared to control cells. Pre- and post-treatment with CBD, significantly decreased (by up to 50%) the negative zeta potential after UVB or hydrogen peroxide.

#### 3.1.2 Penetration of endogenous and exogenous compounds through cell membranes

**Fig. 5:**
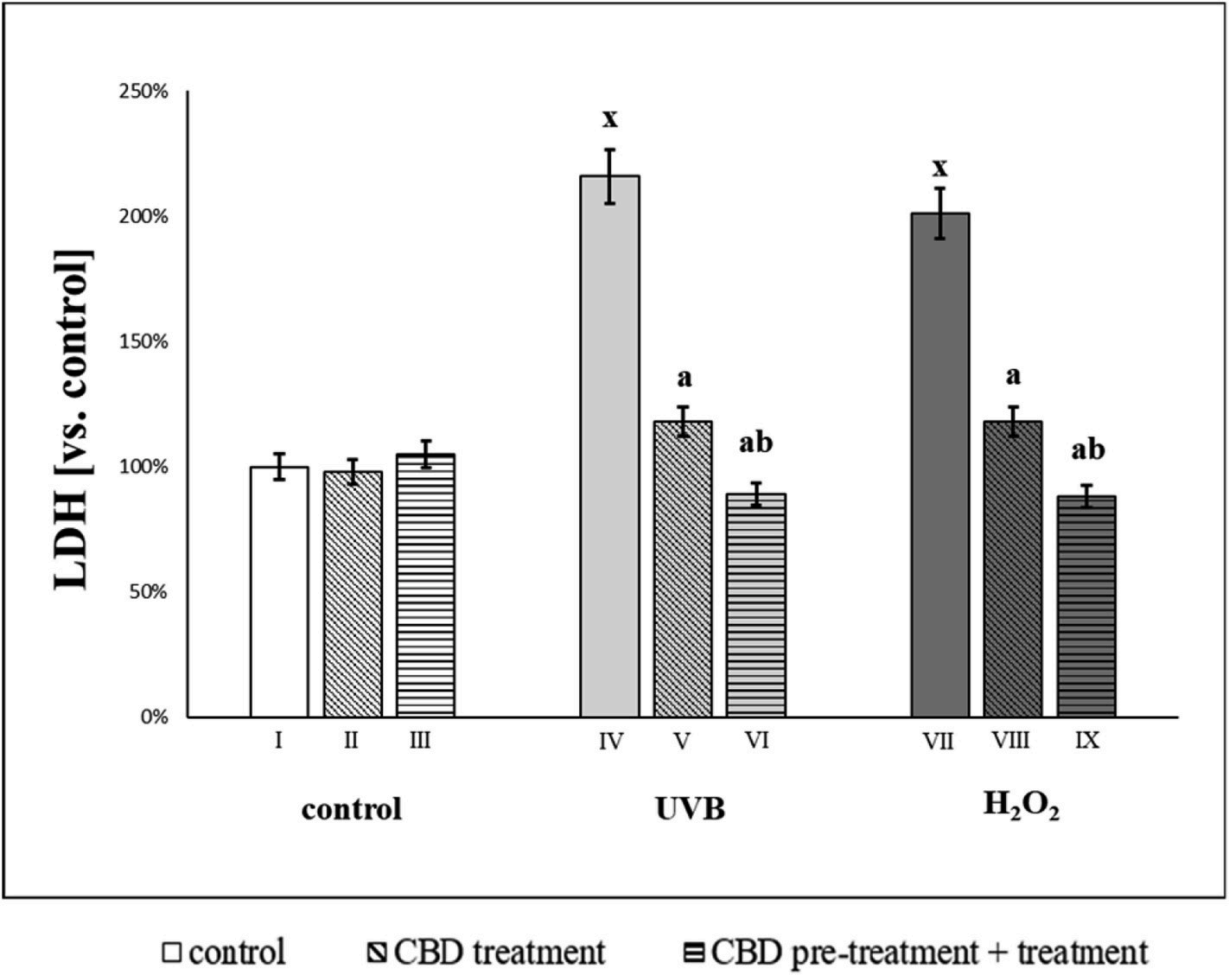
Lactate dehydrogenase (LDH) activity in mediums of keratinocytes: **I.** control; **II.** cultured with CBD (4uM) for 24h; **III**. cultured with CBD (4uM) for 48h; **VI.** irradiated with UVB [60 mJ/cm^2^]; **V.** irradiated with UVB [60 mJ/cm^2^] and cultured with CBD (4uM) for 24h after irradiation; **VI.** irradiated with UVB [60 mJ/cm^2^] and cultured with CBD for 24h befor irradiation and 24h after irradiation; **VII.** exposed to H_2_O_2_ [200μM]; **VIII.** exposed to H_2_O_2_ [200μM] and cultured with CBD (4uM) for 24h after exposer to H_2_O_2_; **IX.** exposed to H_2_O_2_ [200μM] and cultured with CBD (4uM) for 24h before and 24h after exposer to H_2_O_2_. Mean values ± SD of five independent experiments and statistically significant differences for p < 0.05 are presented: a - differences vs. group I/group IV/group VII; b - differences vs. group II/group V/group VIII; x - differences between group IV/group VII and group I.

Changes in the composition of the cell membranes of keratinocytes exposed to irradiation or a chemical agent also affected cell function. We observed that after UVB or hydrogen peroxide keratinocyte membrane permeability increased by a factor of 2, based on LDH leakage from cells. The use of CBD after both irradiation and chemical exposure reduced this leakage by about 50%. However, the use of CBD before and after the above-mentioned factors further increased the protective effect of CBD. It can therefore be concluded that CBD reduced the degree of damage to the keratinocyte membrane, most effectively before and after the exposure to irradiation and chemical factors (Fig. 5).

**Fig. 6:**
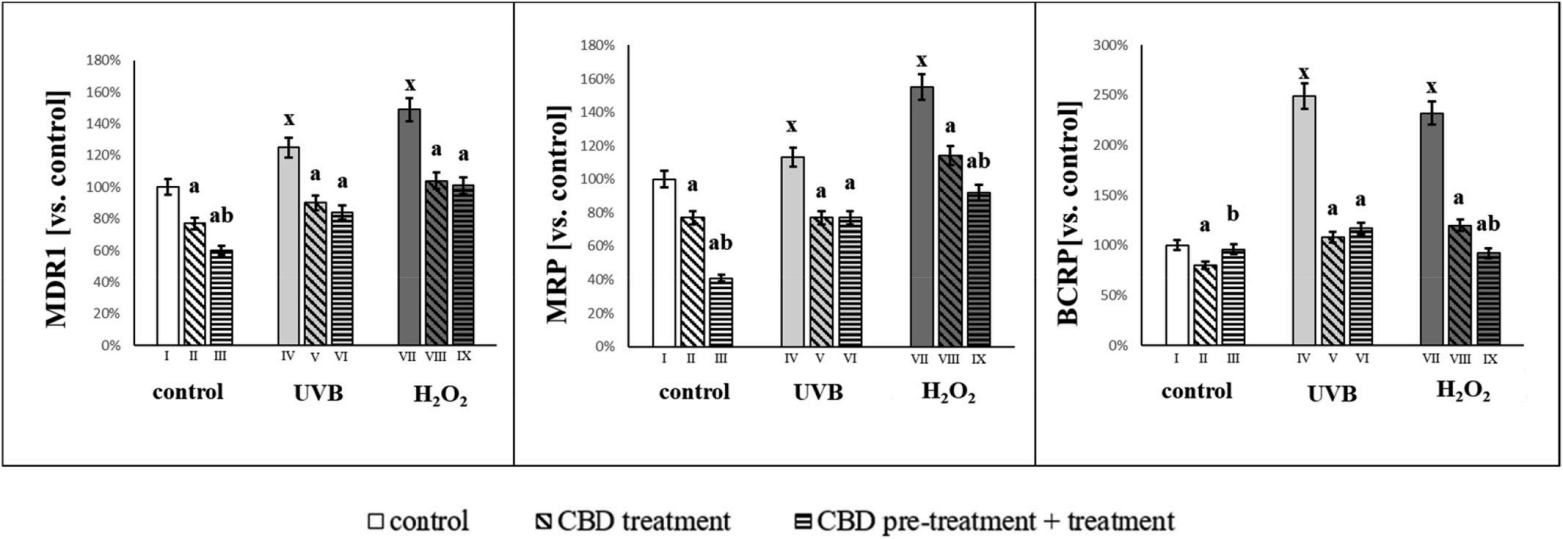
Activities of ABC-cassette transporters (MDR1, MRP, BCRP) in groups of keratinocytes: **I.** control; **II.** cultured with CBD (4uM) for 24h; **III**. cultured with CBD (4uM) for 48h; **VI.** irradiated with UVB [60 mJ/cm^2^]; **V.** irradiated with UVB [60 mJ/cm^2^] and cultured with CBD (4uM) for 24h after irradiation; **VI.** irradiated with UVB [60 mJ/cm^2^] and cultured with CBD for 24h befor irradiation and 24h after irradiation; **VII.** exposed to H_2_O_2_ [200μM]; **VIII.** exposed to H_2_O_2_ [200μM] and cultured with CBD (4uM) for 24h after exposer to H_2_O_2_; **IX.** exposed to H_2_O_2_ [200μM] and cultured with CBD (4uM) for 24h before and 24h after exposer to H_2_O_2_. Mean values ± SD of five independent experiments and statistically significant differences for p < 0.05 are presented: a - differences vs. group I/group IV/group VII; b - differences vs. group II/group V/group VIII; x - differences between group IV/group VII and group I.

After UVB or hydrogen peroxide exposure the activity of ABC cassette transporters (MDR1, MRP, BCRP) increased significantly (Fig. 6). The use of CBD significantly reduced the activity these transporters. After UVB irradiation, CBD treatment decreased the activity of MDR1, MRP and BCRP to 33%, 32% and 56% (vs values of control UVB irradiated groups), respectively. CBD treatment of cells exposed to hydrogen peroxide reduced the activity of MDR1, MRP and BCRP by approximately 32%, 40% and 60% (compared to the values of the groups exposed to hydrogen peroxide), respectively. CBD treatment pre- and post-exposure of keratinocytes to hydrogen peroxide was more effective at reducing MRP and BCRP transporters activity.

**Fig. 7:**
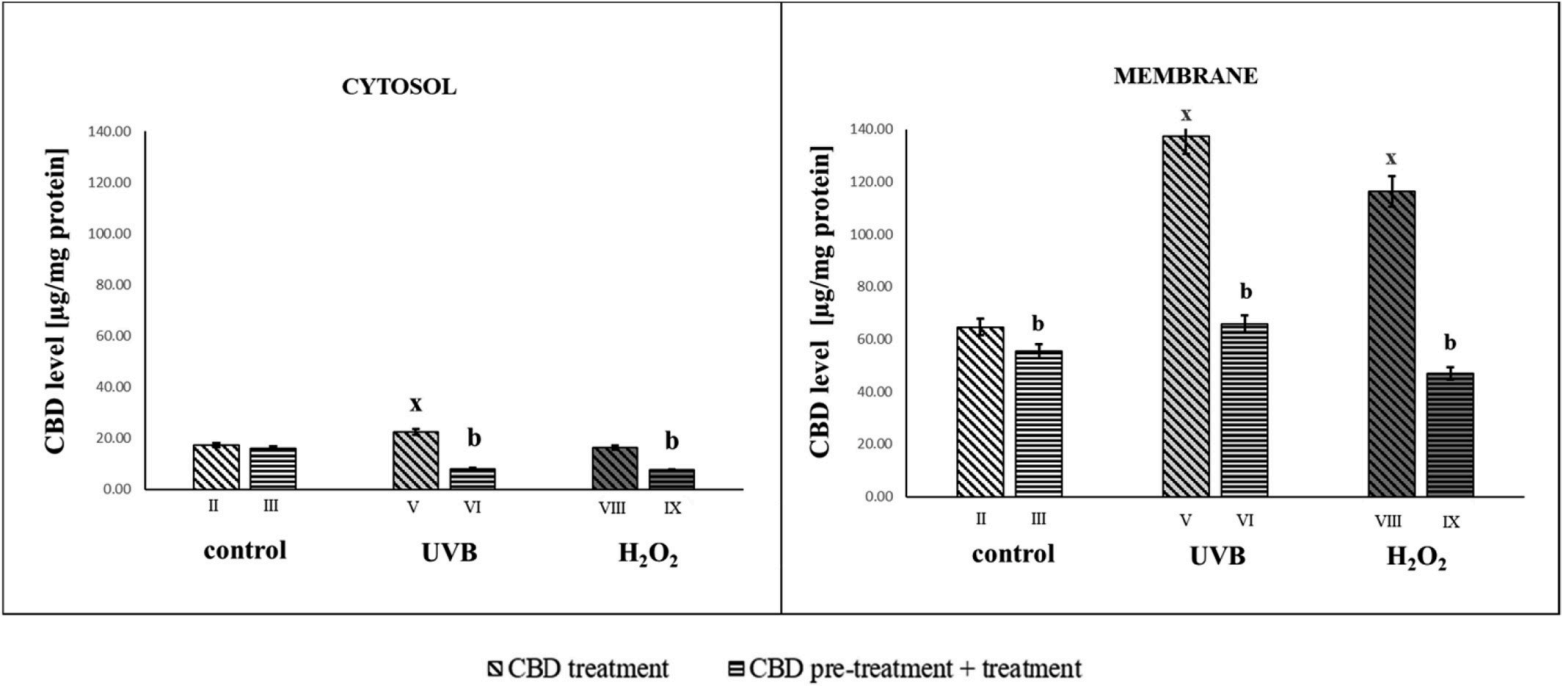
CBD level [μg/mg protein] in groups of keratinocytes: **II.** cultured with CBD (4uM) for 24h; **III**. cultured with CBD (4uM) for 48h; **V.** irradiated with UVB [60 mJ/cm^2^] and cultured with CBD (4uM) for 24h after irradiation; **VI.** irradiated with UVB [60 mJ/cm^2^] and cultured with CBD for 24h befor irradiation and 24h after irradiation; **VIII.** exposed to H_2_O_2_ [200μM] and cultured with CBD (4uM) for 24h after exposer to H_2_O_2_; **IX.** exposed to H_2_O_2_ [200μM] and cultured with CBD (4uM) for 24h before and 24h after exposer to H_2_O_2_. Mean values ± SD of five independent experiments and statistically significant differences for p < 0.05 are presented: b - differences vs.group II/group V/group VIII; x - differences between group IV/group VII and group I.

It can therefore be concluded that oxidative stress induced in keratinocytes by UVB irradiation or hydrogen peroxide not only contributed to the modification of phospholipids of the keratinocytes membrane, but also affected membrane proteins (such as transmembrane ABC transporters) that may participate in the supply/removal of exo- and endogenous compounds. Increased activity of ABC membrane transporters was associated with greater membrane permeability allowing CBD to enter the cytosol more efficiently following UVB irradiation (Fig. 7).

## 4 Discussion

The skin is the main barrier which provides protection against damaging environmental factors. Among these, the most common is sunlight which is particularly important due to high energy UVB radiation, which disturbs the metabolism of keratinocytes [41]. Chemical factors can also affect cutaneous biology, by altering cellular membrane composition, structure, and functions [42].

Skin cells are characterized by active and diverse lipid metabolism, which is necessary for barrier and signaling functions [43]. The skin's functions are supported by cell membrane proteins, whose biological activities promote the proper interactions of cells with each other and with the environment [44]. Therefore, metabolic alterations caused by external factors that can lead to oxidative stress and oxidative modifications of cellular components may cause changes in membrane structure and biological activity. This can have downstream effects on cellular metabolism, leading to functional skin cell disorders and ultimately contributing to the development of numerous skin diseases such as erythema, photoallergic reactions, autoimmune diseases, psoriasis, neutrophilic disorders, and cancer [45].

CBD, a compound with known anti-inflammatory and antioxidant properties [23,46], could be considered as a potential therapeutic compound to protect against the damaging environmental factors. The evidence presented here supports this notion, as the results indicate that CBD effectively prevents changes in the structure and function of cell membranes of keratinocyte exposed to UVB irradiation or hydrogen peroxide.

The results of this study confirm earlier reports indicating that hydrogen peroxide, and in particular UVB irradiation, contributes to the production of superoxide radicals and reduces the antioxidant capacity of keratinocytes. Mechanistically, this is through increasing the activity of xanthine oxidase and NADPH, [47]. In contrast, the use of CBD counteracts redox imbalances caused by UVB irradiation and hydrogen peroxide in human keratinocytes, both at the level of oxidants and antioxidants. Earlier literature also pointed to the protective role of CBD, but in other pathological conditions [48,49]. Based on the literature, it could be suggested that this effect results from the CBD phenolic structure that provides the ability to terminate free radical chain reactions, capture free radicals, or convert radicals to less active forms [50]. CBD also reduces the generation of ROS by chelating the transition metal ions involved in the Fenton reaction to form extremely reactive hydroxyl radicals [51]. In addition to directly reducing the level of oxidants, CBD also modifies the redox balance by changing the level and activity of endogenous antioxidants [23].

CBD may also modify other metabolic pathways in keratinocytes that alleviate disorders resulting from UVB and hydrogen peroxide exposure. It has been previously shown that UVB irradiation can modify various molecular signals transmitted via phosphorylation of JNK and AKT kinases [52], while hydrogen peroxide affects redox signaling, and modulates the activity of transcription factors, including Nrf2, which is responsible for transcription of cytoprotective genes [[53], [54], [55]]. It has recently been observed that this effect occurs as a result of inhibiting the expression of Bach1 - the regulator of transcription factor Nrf2 - and thereby increasing the expression of Nrf2 target genes such as hemeoxygenase 1 [26]. The changes caused by CBD in the level/activity of cytoprotective proteins result not only from modifications at the mRNA level, but also at the protein level. It is known that UVB and hydrogen peroxide modify the functions of proteins by oxidizing biologically important protein thiol groups [56,57], while CBD - in direct interaction with cysteine - can reduce glutathione or protein disulfide bridges, and thus restore their biological activity [58]. CBD can simultaneously modify the interaction of components of the metabolic pathways of Nrf2 and nuclear factor-κB (NF-κB), and thus simultaneously affect the antioxidant and anti-inflammatory abilities of cells [27].

The changes in ROS levels and antioxidant capabilities presented here, suggest that UVB irradiation disturbs the redox balance more strongly than hydrogen peroxide, which would indicate that the severity of keratinocyte oxidative stress is also greater after UVB irradiation. However, the analysis of the effects of oxidative stress assessed by changes in the composition/structure and function of biological membranes suggests that both UVB and hydrogen peroxide clearly, but distinctly, modify the lipid structure of the membranes.

It is known that oxidative stress associated with UVB irradiation and hydrogen peroxide exposure promotes oxidative modifications of cellular components, including phospholipid polyunsaturated fatty acids [59,60]. This is especially visible in the case of UVB irradiated keratinocytes, which have increased phospholipid oxidative fragmentation and cyclization, demonstrated by an increase in MDA and F_2_-isoprostane levels. As a result, UVB irradiation significantly reduced the level of phospholipid polyunsaturated fatty acids. Hydrogen peroxide also increased the oxidative cyclization of PUFA's hydrocarbon chains, indicated by an increase in F_2_-isoprostane levels. However, since an increase in the level of PUFA's has been observed after the use of hydrogen peroxide [61], the increase in the level of these acids, together with the enhanced lipid peroxidation process, may be a consequence of the intensification of PUFA's *de novo* synthesis in response to metabolic changes caused by exogenous factors.

Another membrane component that undergoes modification during oxidative stress is sialic acid, which is part of glycolipids and glycoproteins. Sialic acid is a carrier of a negative charge on the surface of the membrane, and therefore changes the physicochemical properties and zeta potential of cell membranes [62,63]. UVB and hydrogen peroxide exposure caused an increase in electric charge on the surface of keratinocyte cells, which corresponded to an increase in sialic acid content and a decrease in cell size. Published data confirm the relationship between the increase in sialic acid level and ROS generation but also altered sialylation caused by UV irradiation [16]. In addition, under the influence of UVB or hydrogen peroxide, the barrier function of epidermal cells is impaired, and intracellular water leaves the keratinocytes by osmosis, which leads to cell shrinkage [64]. Maintaining a constant cell volume is critical to normal cell activity, such as growth, migration, and regulation of intracellular metabolism [65,66]. CBD treatment reduced the damaging effects of UVB and hydrogen peroxide, and helped restore proper regulation of keratinocyte metabolism.

CBD treatment significantly prevented membrane phospholipid responses to the exogenous factors. This was evident both in the level of phospholipid PUFAs, lipid peroxidation products (MDA and F_2_-isoprostanes), as well as in the zeta potential and cell size. The consequence of decreasing changes in the structure of membranes is a significant reduction in membrane permeability, which was assessed on the basis of LDH levels in the extracellular space. Since CBD belongs to lipophilic antioxidants, its main sites of action are biological membranes. This was confirmed by the results of this study, as an accumulation of CBD was observed in the keratinocyte layer. Therefore, CBD protects biomembranes, preventing phospholipid peroxidation. Earlier studies have shown that - regardless of protection against changes caused by the physicochemical effects of exogenous factors - CBD treatment also reduces MDA levels in mouse hippocampal neuronal cell [67], as well as in the heart tissue of Sprague-Dawley rats [68]. In addition, a similar trend of changes in level of lipid peroxidation product (MDA and 4-HNE) was observed in fibroblasts after using other lipophilic antioxidants such as rutin [3,69].

Changes in the structure of the cell membrane may also include modifications of membrane proteins, which can undergo oxidative modifications as a result of reaction with ROS and lipid peroxidation products. This may also apply to membrane transporters (ABC) carrying exogenous substances and their metabolites across membranes. Accordingly, UVB and hydrogen peroxide-induced oxidative stress enhance the activity of transmembrane transporters (MRP, MDR and BCRP), but CBD treatment impedes this activity. Previous reports confirm that the expression of ABC transporters, including MDR1, MRP1, MRP2, MRP4, and BCRP, increases as a result of oxidative stress [70]. Studies on mouse brain cells have shown that knocking out MDR1 or BCRP in transgenic animals does not affect CBD transport, while CBD inhibits both MDR1 and BCRP transporters [71]. In humans, CBD also inhibits the activity of MRP1 in ovarian cancer cells [72], while inducing BCRP expression and reducing MDR1 expression in human trophoblast-like cell lines, BeWo and Jar [73]. CBD, which activates redox-sensitive transcription factor - Nrf2 [27], can thus induce MRP expression as shown in C57BL/6J mouse hepatocytes [74]. Activating Nrf2 also induces the expression and transport function of MDR1, MRP2, and BCRP in the blood-brain and blood-spinal cord barriers of Sprague Dawley rats [75]. However, to increase the expression/activity of transporters by Nrf2, the contribution of additional factors (p53, p38 and NFκB) is necessary [75].

Based on the above data, it is difficult to determine whether and what transporters are involved in the transmission of CBD across cell membranes. However, it is certain that CBD has an inhibitory effect on the activity of these transporters, particularly after UVB irradiation. In addition, it is not known whether its structure is modified during transport of CBD across membranes, which may also promote inhibition of transporter activity. This may also be the reason that in our studies we found significantly lower CBD levels in the cytosol than in membranes, especially in longer treated cells. In addition the level of cytosol free CBD may be decreased by its interaction with fatty acid-binding proteins (FABPs), which mediate anandamide transport to its catabolic enzyme fatty acid amide hydrolase but is also target for CBD [76]. In addition to above, active molecules in the membrane and cytosol, including ROS and lipid peroxidation products may interact with membrane transporters. Moreover the transport of various compounds across cell membranes is also dependent on the sialic acid contained in glycoproteins and membrane glycolipids, the levels of which change under the influence of exogenous factors, as indicated above. Regarding CBD penetration through cell membranes, recent studies also suggest that CBD may interact directly with voltage-dependent anion channels (VDACs) in the outer mitochondrial membrane [77]. VDACs have also been observed in the cell membrane of several cell lines including lymphocytes, epithelial cells, and astrocytes [78]. However, CBD-inducing VDAC1 channel closure may decrease CBD level in keratinocytes [79]. In addition, it should be taken into account that structural and metabolic changes related to CBD activity are exacerbated by the oxidative action of UVB or hydrogen peroxide and can then be further modified as a result of CBD re-action. The results obtained here do not allow to identify changes caused by secondary interactions. In addition, it has previously been shown that cytoprotective compounds can have different time-dependent effects [80]. Therefore, given the two ways of CBD administration (after UVB radiation as well as before and after UVB radiation), it is possible that CBD interactions with cell components may also be modified. It is known that CBD undergoes oxidative metabolism and its concentration in biological material decreases over time [81]. In this, decarbonylation has been observed to form DCBD, a compound with reduced hydrophilic properties and longer retention time relative to CBD (under LCMS analysis), shown in human samples [82].

It should be taken into account that structural and metabolic changes after CBD pretreatment can be modulated by the oxidative action of UVB or hydrogen peroxide and can then be further modified as a result of CBD re-action. The results obtained here do not allow to identify changes caused by secondary interactions. Therefore, the results obtained in this work require further qualitative and quantitative analysis.

## 5 Conclusion

CBD reduces the harmful effects of UVB and hydrogen peroxide on keratinocytes, which is associated with CBD's antioxidant activity. This phytocannabinoid, by regulating the redox balance of cells, partially prevents oxidative changes affecting phospholipids, proteins and sialic acid in keratinocyte membranes, which helps maintain membrane integrity. CBD regulates the activity of ABC membrane transporters. Due to its beneficial protective effect, CBD can be disclosed as a protective agent for skin keratinocytes against the harmful effects of radiation and chemical environmental factors that cause oxidative stress in skin cells.

## Declaration of competing interest

The authors have no conflicts of interest to declare.
